# Simulated Target Attainment of Multidose Regimens of Dalbavancin for Prolonged Durations of Therapy

**DOI:** 10.1093/ofid/ofae315

**Published:** 2024-06-06

**Authors:** Cecilia F Volk, Paul R Hutson, Warren E Rose

**Affiliations:** Pharmacy Practice and Translational Research Division, School of Pharmacy, University of Wisconsin–Madison, Madison, Wisconsin, USA; Pharmacy Practice and Translational Research Division, School of Pharmacy, University of Wisconsin–Madison, Madison, Wisconsin, USA; Pharmacy Practice and Translational Research Division, School of Pharmacy, University of Wisconsin–Madison, Madison, Wisconsin, USA

**Keywords:** bacteremia, dalbavancin, osteomyelitis, pharmacokinetic

## Abstract

**Background:**

Dalbavancin is a long-acting lipoglycopeptide antibiotic that is increasingly utilized for infections that require prolonged treatment durations despite the lack of Food and Drug Administration approval for these indications. There is no consensus regarding optimal dosing of dalbavancin for these infections and no available pharmacokinetic studies to identify optimal dosing for long-term use.

**Methods:**

An in silico pharmacokinetic simulation was performed to assess the predicted dalbavancin concentration resulting from commonly utilized dosing regimens, in addition to modified regimens. The primary endpoint evaluated was days of median 24-hour free area under the curve over the minimum inhibitory concentration (AUC/MIC) >27.1, the established PK target.

**Results:**

A dosing regimen of 1500 mg on day 0 and day 7 resulted in median AUC/breakpoint value above the target for 57 days (lower 95% confidence interval [CI], 37 days). A modified regimen of 1500 mg on day 0 and day 21 resulted in an additional 11 days of median AUC/breakpoint target attainment. The other standard dosing regimen modeled was 1000 mg on day 0, then 500 mg weekly for 5 doses. This regimen achieved the AUC/breakpoint target for 76 days (lower 95% CI, 59 days). This regimen was modified to 1000 mg on day 0, then 500 mg on days 14 and 28, which shortened the median effective treatment duration by 14 days but required 3 fewer doses.

**Conclusions:**

These simulated results, when combined with the favorable observational data, support the use of commonly reported dalbavancin regimens for prolonged therapy durations. In addition, these pharmacokinetic/pharmacodynamic data support extending the dosing interval beyond the frequently reported weekly regimens, which should be investigated further with a clinical trial.

## Teaser

Dalbavancin regimens frequently utilized for prolonged therapy durations were assessed using in silico pharmacokinetic simulations. The resulting predicted serum concentrations support the use of dalbavancin for prolonged therapy and indicate that dosing intervals longer than weekly could be utilized.

The management of multidrug-resistant gram-positive infections such as methicillin-resistant *Staphylococcus aureus* is often a complicated task for clinicians. These infections are even more difficult when prolonged treatment durations are required, such as for bacteremia, endocarditis, or osteomyelitis. While many treatment options exist, barriers such as intravenous access, outpatient infusion coordination, and insurance coverage often complicate management. The use of long-acting lipoglycopeptides (LGPs) has become an attractive option for clinicians and patients who wish to utilize intravenous therapy with less frequent dosing regimens and avoid the placement of central catheters.

Dalbavancin (DAL) is one of 2 LGPs that have been utilized for these complicated infections. It is Food and Drug Administration–approved for the treatment of skin and soft tissue infections caused by susceptible gram-positive organisms [1]. There are 2 approved dosing regimens for this indication: (*i*) 1500 mg given as a single dose and (*ii*) 1000 mg followed by 500 mg 1 week later. Both have been evaluated in randomized controlled trials (RCTs) and demonstrated noninferiority to standard of care [2, 3].

Off-label use of DAL has also become quite common. Specifically, many clinicians have begun to utilize DAL for infections that require prolonged durations of therapy, including bacteremia, osteomyelitis, and endocarditis. This is a potentially attractive treatment option in patients who are unwilling or unable to maintain a peripherally inserted central line or remain hospitalized for the duration of therapy. A single RCT exists supporting the use of DAL for osteomyelitis, which utilized a regimen of 1500 mg weekly for 2 doses [4]. In addition, there are a number of observational reports of DAL use reporting favorable outcomes for prolonged durations of therapy [5–14]. These studies have utilized a wide variety of dosing regimens, with the 2 most commonly reported as either 1500 mg weekly for 2 doses (as studied in the available RCT) or 1000 mg followed by 500 mg weekly for variable duration (up to 32 weeks reported) [8].

In this study, we used population pharmacokinetic (PK) estimates to evaluate these 2 most common dosing strategies that have been reported for DAL and assessed the serum antibiotic concentrations for attainment of the identified pharmacodynamic target for DAL.

## METHODS

An in silico simulation was performed to assess the predicted DAL concentrations that would result from a variety of dosing regimens. The population PK used was developed by Carrothers et al, which utilized PK sampling data from 4 safety and efficacy studies [3, 15–18]. The resulting model had 3-compartment distribution and first-order elimination with a total volume of distribution of approximately 15 L and a clearance of 0.05 L/hour. The covariate relationships included in the final simulation were age, albumin, creatinine clearance, and weight. Ninety-three percent protein binding was assumed [1]. A summary of this PK model can be found in the Supplementary Materials, with full details of covariate relationships available in the original publication [15].

A dataset of 1000 hypothetical patients was generated with ages between 20 and 80 years, serum albumin concentrations between 2.5 and 4.5 g/dL, creatinine clearance between 30 and 120 mL/minute, and body weights between 40 and 200 kg. In the construction of the 1000 simulated patients, the demographics that served as covariates in the model were randomly sampled with replacement, leading to approximately equal frequencies of each value among the 1000 simulated patients and random assignment of the possible values of the 4 parameters. Table 1 describes the distribution of these covariates. NONMEM (v7.5, ICON, Dublin, Ireland) was used to simulate free plasma concentrations for these 1000 hypothetical patients for each modeled regimen using the established model published by Carrothers et al [15]. The complete NONMEM control file data can be found in the Supplementary Materials.

**Table 1.** Inclusion of Covariates Within Model Simulations

Covariate	Range	Increment	Mean (SD)
Weight, kg	40–200	10	120.74 (49.1)
Albumin, g/dL	2.5–4.5	0.25	3.5 (0.7)
Age, y	20–80	10	50.9 (19.6)
CrCl, mL/min	30–120	10	76.5 (28.5)

Abbreviations: CrCl, creatinine clearance; SD, standard deviation.

The free 24-hour area under the curve over the minimum inhibitory concentration (24-hour *f*AUC/MIC), which has an established target for stasis of 27.1 based on a neutropenic murine thigh infection model, was assessed [19]. The primary PK/pharmacodynamic (PD) target assessed was the number of days with the 24-hour *f*AUC/MIC above the target for each dosing regimen. The 24-hour *f*AUC was estimated for each individual day of the simulated treatment period. Two MIC benchmarks were used: (*i*) the breakpoint of 0.25 µg/mL established by the Clinical and Laboratory Standards Institute (CLSI) and (*ii*) the 90^th^ percentile MIC (MIC_90_) (0.06 µg/mL) for *S aureus* reported from worldwide surveillance studies for DAL [20–22]. Given a preferred target 24-hour ratio *f*AUC/MIC = 27.1, the target 24-hour *f*AUC for an organism at the breakpoint is 6.8 µg × hour/mL and for the MIC_90_ is 1.6 µg × hour/mL. In addition, the secondary outcome of duration of time (days) where the free serum concentration was expected to be above the MIC (T > MIC) was also assessed.

## RESULTS

**Figure 1.**
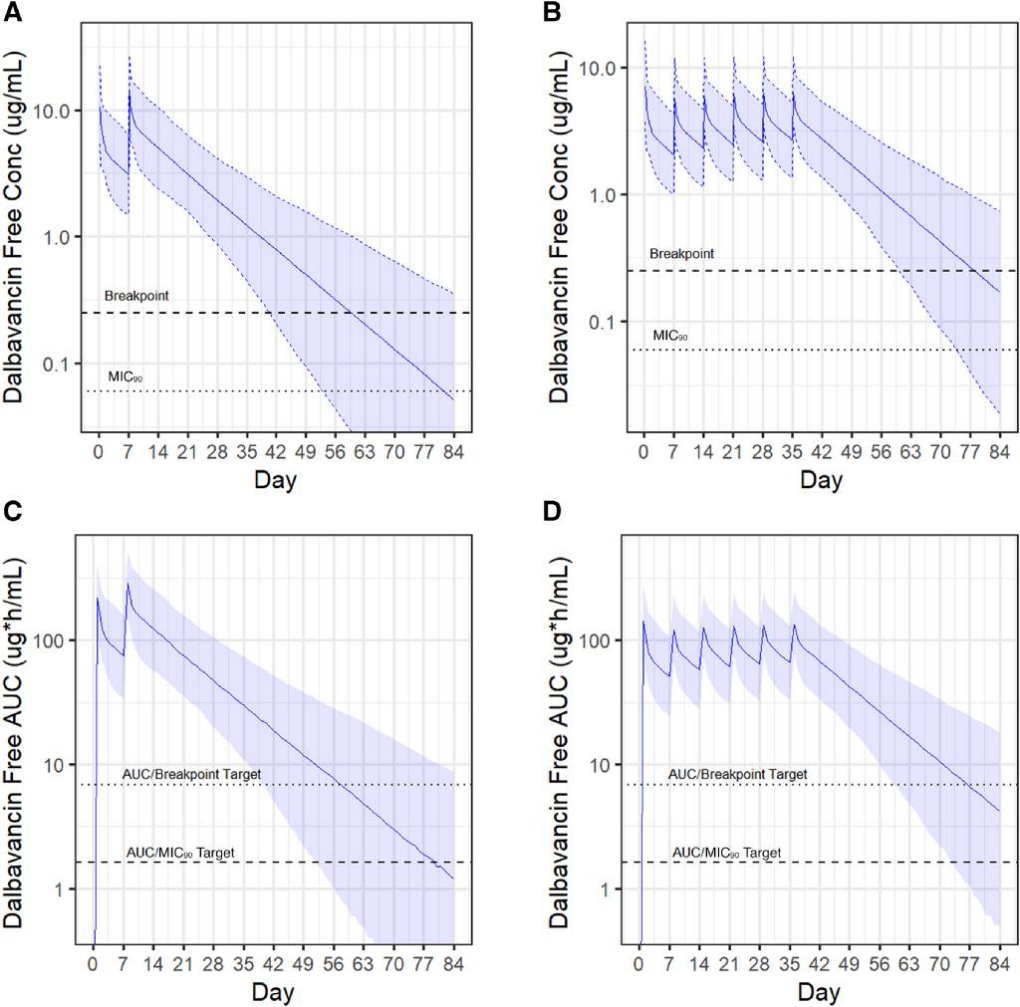
Simulated dalbavancin (DAL) exposure after standard DAL regimens. Solid lines indicate the median value with shaded regions highlighting the 95% confidence interval. *A* and *B*, Free serum DAL concentrations after administration of DAL 1500 mg on day 0 and day 7 (*A*) or DAL 1000 mg on day 0, then 500 mg weekly for 5 weeks (*B*). The breakpoint (0.25 mg/L) and 90^th^ percentile MIC (MIC_90_) (0.06 mg/L) are indicated by dashed lines. *C* and *D*, 24-h free area under the curve (AUC) after administration of DAL 1500 mg on day 0 and day 7 (*C*) or DAL 1000 mg on day 0, then 500 mg weekly for 5 weeks (*D*). Both AUC/MIC targets (6.8 for the breakpoint and 1.6 for the MIC_90_) are indicated by dashed lines.

The 2 most reported prolonged DAL dosing regimens were modeled in silico with predicted free serum concentrations and 24-hour *f*AUC values shown in Figure 1. The regimen of 1500 mg on day 0 and day 7 maintained values above the 24-hour *f*AUC/MIC_90_ target for a median of 79 days (lower 95% confidence interval [CI], 52 days) and above the 24-hour *f*AUC/breakpoint target for a median of 57 days (lower 95% CI, 37 days) (Table 2). The regimen of 1000 mg on day 0 followed by 500 mg weekly for 5 doses led to overall longer effective treatment duration, with values above the 24-hour *f*AUC/MIC_90_ for a median of 98 days (lower 95% CI, 73 days) and 24-hour *f*AUC/breakpoint target for a median of 76 days (lower 95% CI, 59 days). Overall, there was nominal difference in time of target attainment when utilizing the secondary PK/PD endpoint of time above the MIC (Table 2).

**Table 2.** Time of Target Achievement for Each Simulated Regimen

Regimen	Days With 24 h *f*AUC/MIC >27.1 (Median [Lower 95% CI])		Days With Free Serum Concentration >MIC (Median [Lower 95% CI])	
	MIC_90_	Breakpoint	MIC_90_	Breakpoint
1500 mg days 0 and 7	79 (52)	57 (37)	81 (53)	59 (40)
1000 mg day 0, 500 mg weekly × 5 doses	98 (73)	76 (59)	99 (73)	77 (60)
1500 mg days 0 and 21	91 (66)	68 (52)	92 (66)	70 (51)
1000 mg day 0, 500 mg days 14 and 28	84 (63)	62 (50)	86 (63)	64 (50)

Abbreviations: CI, confidence interval; *f*AUC/MIC, free 24-hour area under the curve over the minimum inhibitory concentration; MIC_90_, 90^th^ percentile MIC.

**Figure 2.**
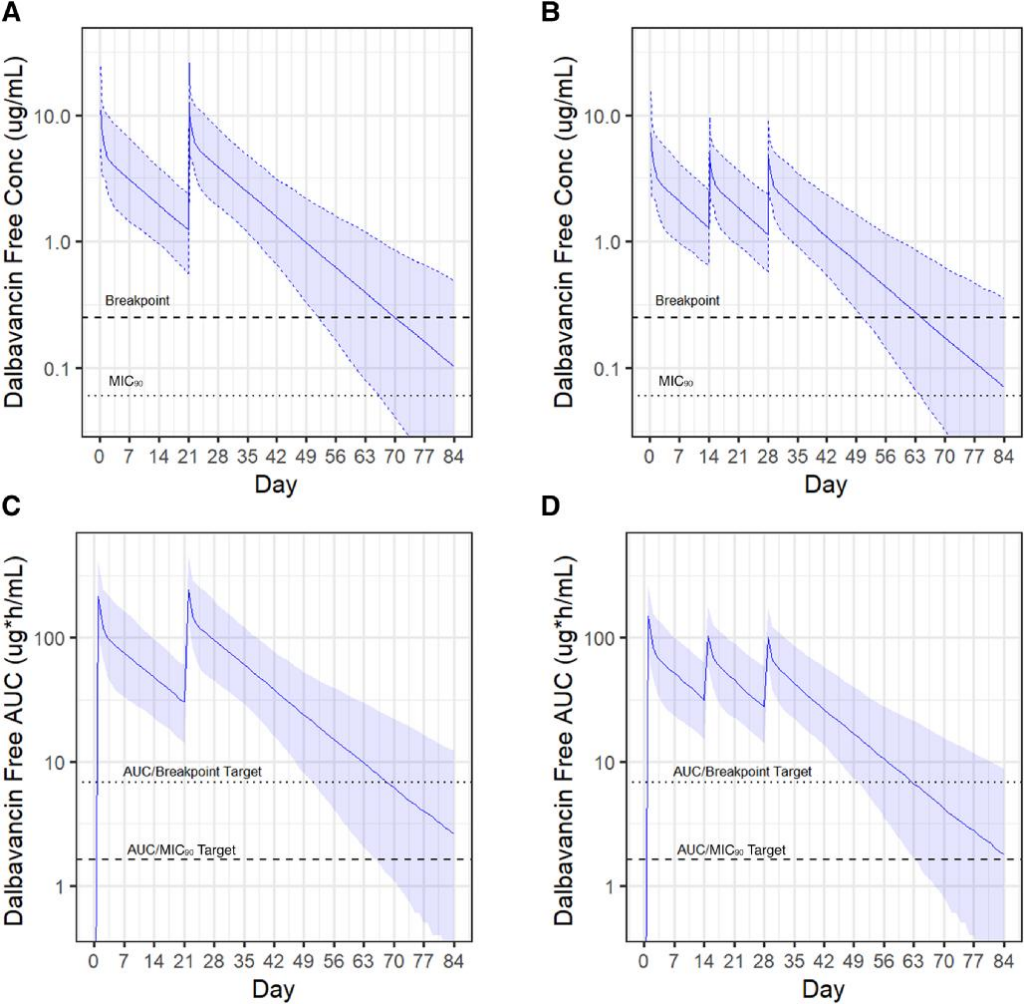
Simulated dalbavancin (DAL) exposure after modified DAL regimens. Solid lines indicate the median value with shaded regions highlighting the 95% confidence interval. *A* and *B*, Free serum DAL concentrations after administration of DAL 1500 mg on day 0 and day 21 (*A*) or DAL 1000 mg on day 0, then 500 mg on days 14 and 28 (*B*). The breakpoint (0.25 mg/L) and 90^th^ percentile MIC (MIC_90_) (0.06 mg/L) are indicated by dashed lines. *C* and *D*, 24-h free area under the curve (AUC) after administration of DAL 1500 mg on day 0 and day 21 (*C*) or DAL 1000 mg on day 0, then 500 mg on days 14 and 28 (*D*). Both AUC/MIC targets (6.8 for the breakpoint and 1.6 for the MIC_90_) are indicated by dashed lines.

Based on the results from modeling these standard dosing regimens, we hypothesized that each regimen could be optimized to achieve similar target attainment with lower and/or fewer doses. These regimens were modeled and analyzed in the same manner, with predicted free serum concentrations and 24-hour *f*AUC values shown in Figure 2. First, to modify the regimen of 1500 mg on day 0 and day 7, we extended the dosing interval and simulated a regimen of 1500 mg on day 0 and day 21. This modification extended the time above the 24-hour *f*AUC/MIC targets by about 11 days (Table 2), allowing a longer effective treatment duration without requiring additional doses to be administered. Of note, the 24-hour *f*AUC and free serum concentration did not fall below target levels between the 2 doses given 3 weeks apart. Second, to modify the regimen of 1000 mg on day 0 followed by 500 mg weekly for 5 doses, we opted to evaluate a regimen that extends the dosing interval and reduces the amount of DAL administered: 1000 mg on day 0 followed by 500 mg on day 14 and day 28. This modification decreased the duration of target attainment by 14 days following the last dose (Table 2), but again, extending the dosing interval did not lead to serum levels below the target between doses.

## DISCUSSION

The complications of the use of the long-acting lipoglycopeptides include the cost of these regimens combined with the relatively few PK data to support a particular regimen. Therefore, evaluation of target attainment and dose modification is of importance to optimize their use for infections requiring prolonged antibiotic therapy. The only RCT utilizing extended dosing of DAL utilized a regimen of 1500 mg on days 0 and 7. Our data show that, while the median concentrations would achieve an 8-week treatment, the simulated 95% CI falls below the AUC target at approximately 5.5 weeks for an isolate at the breakpoint, indicating that some patients may receive a shorter treatment duration based on the model covariates used. To extend the treatment duration without increasing the total amount of drug administered, we decided to evaluate a modified DAL regimen of 1500 mg on days 0 and 21. This dosing regimen is expected to provide a longer effective treatment duration (for the entire 95% CI) than the standard regimen by about 11 days. Neither the serum concentration nor the AUC fell below the targeted levels before the second dose was administered on day 21. This dosing regimen therefore makes it possible to increase the total treatment time without requiring additional doses and could be an important cost-saving measure for patients who require prolonged therapy.

The other common DAL dose used off label for prolonged treatment is 1000 mg on day 0 followed by 500 mg weekly. This regimen achieved treatment durations well beyond 8 weeks for all patients within the 95% CI. Similar to the other standard regimen, it did not appear necessary to administer DAL doses as close together as weekly. Therefore, to reduce drug and administration cost (as well as the number of visits required for a patient), we evaluated a modified regimen of 1000 mg on day 0, then 500 mg on days 14 and 28, which would only require 3 doses total (vs 6 doses for the standard regimen). Although this modified regimen eliminates 3 doses, it also shortens the time above the AUC targets. With this regimen, the modeled parameters suggest target attainment (median days above *f*AUC/breakpoint target) was shortened by about 14 days. Based on the model, this regimen would require another 500-mg dose given on day 42, to ensure adequate therapy (based on target attainment) for an ≥8-week duration of therapy if required for a specific patient. We note that the AUC/breakpoint is a more conservative estimate of target attainment compared to AUC/MIC_90_ since the MIC_90_ is a 4-fold lower threshold. Therefore, this additional dose may not be required for target attainment for isolates below the breakpoint MIC of 0.25 µg/mL. It is also important to note that the consequences of missing a dose in this 3-dose regimen are likely more severe than missing a dose of a planned 6-dose regimen. Because of this, some may prefer to plan for a 6-dose regimen in certain patient populations with adherence concerns.

While this study reports serum DAL concentrations, it is important to consider penetration into bone and other tissues when treating deep-seated infections such as osteomyelitis. To our knowledge, there is only 1 study (which included 31 patients) that measured the bone concentration of DAL [23]. This study demonstrated an average bone/plasma AUC penetration ratio of 13.1%. The authors conclude that predicted bone concentrations are similar to free serum concentrations (considering 93% protein binding) and that the regimen 1500 mg on days 0 and 7 is expected to maintain unbound dalbavancin concentrations >0.12 µg/mL (the MIC_99.9_) for an 8-week duration. There are several limitations to this study, including the low sample size and overall weak fit of the data to the final model. In addition, the included patients were healthy volunteers and the authors noted that drug penetration may be higher in patients with infected bone. Although our study used a 3-compartment distribution model, we opted not to presume bone penetration in this study due to lack of plasma/bone data in the previously reported 3-compartment PK model and the lack of an established PK/PD target for DAL in bone.

The patients included in this simulation resemble the patients included in the Carrothers et al study, which limits extrapolation to other populations [15]. The covariates from this prior study included albumin, age, creatinine clearance, and weight with an assumed 93% DAL protein binding. Our modeled patients included a range (as described above) for each of these variables, greatly increasing the translatability of these model results. These estimates should be interpreted with caution, however, for patients with significant deviations from these values, although DAL dose reduction is only recommended in patients with significant renal impairment (creatinine clearance <30 mL/minute) [1]. Fortunately, the PK studies included from the previous model included obese patients, with weights ranging from 43 to 320 kg. Another study modeled DAL exposure in a 75-kg individual compared to a 225-kg individual and found only a 33% reduction in total AUC, despite a 3-fold difference in weight [24]. This study did not recommend dose adjustments of DAL for obese patients, which is supported by the limited clinical data available in this population [25]. In addition, it is worth noting that our study used population-based MIC estimates including the CLSI breakpoint and the MIC_90_ for *S aureus*, so application of these results to patients with other organisms or *S aureus* MICs above these thresholds should be used with caution.

In summary, the results of our study support new dosing paradigms for prolonged DAL treatment to increase the time between doses while maintaining effective therapeutic concentrations over 6–8 weeks. This includes either alternative regimen of 1500 mg on days 0 and 21, or 1000 mg on day 0 then 500 mg on days 14 and 28. Based on these simulations, the serum concentrations and AUC values of these regimens should improve PK/PD target attainment in these extended dosing intervals, with no risk of subtherapeutic levels between doses. It is important to note, however, that until clinical data supporting these dosing regimens are available, these modified regimens should be utilized with caution. The specific dosing regimen that should be utilized for a patient must consider the required treatment duration (per the indication), the patient's ability to receive the infusion, and insurance coverage. The lack of clinical trials to support the use of DAL for prolonged durations of therapy is a significant limitation to its application for these clinical situations. Further patient PK/PD and clinical trial efficacy data from such extended-interval treatment paradigms are warranted.

## Supplementary Data

Supplementary materials are available at *Open Forum Infectious Diseases* online. Consisting of data provided by the authors to benefit the reader, the posted materials are not copyedited and are the sole responsibility of the authors, so questions or comments should be addressed to the corresponding author.

## Supplementary Material

ofae315_Supplementary_Data

## Notes

***Author contributions*.** C. F. V.: Data analysis and interpretation, manuscript preparation and revisions. P. R. H.: Experimental design, data analysis and interpretation, revision of manuscript drafts. W. E. R.: Conceptualization, data analysis and interpretation, revision of manuscript drafts.

***Financial support*.** This work was supported by internal funding at The University of Wisconsin–Madison, School of Pharmacy.
