# Supplementary material for: Simulated Target Attainment of Multidose Regimens of Dalbavancin for Prolonged Durations of Therapy
Source: Open Forum Infect Dis. 2024 Jun 6;11(6):ofae315. doi: 10.1093/ofid/ofae315 (PMC11210303; doi:10.1093/ofid/ofae315)
Supplement: ofae315_Supplementary_Data [file ofae315_supplementary_data.zip › Dalba PK - Supplement.docx]

**Simulated Target Attainment of Multi-Dose Regimens of Dalbavancin for Prolonged Durations of Therapy**

Cecilia Volk, Paul Hutson, Warren Rose

**Supplemental Materials**

**Covariate inclusion**:

The model developed by Carrothers et al. included the following covariate relationships:

| **Model Parameter** | **Covariate Relationships** |
| --- | --- |
| CL | CrCl, weight, albumin |
| V1 | Weight, albumin |
| V2 | Weight, albumin, age |
| V3 | Weight, albumin |

**
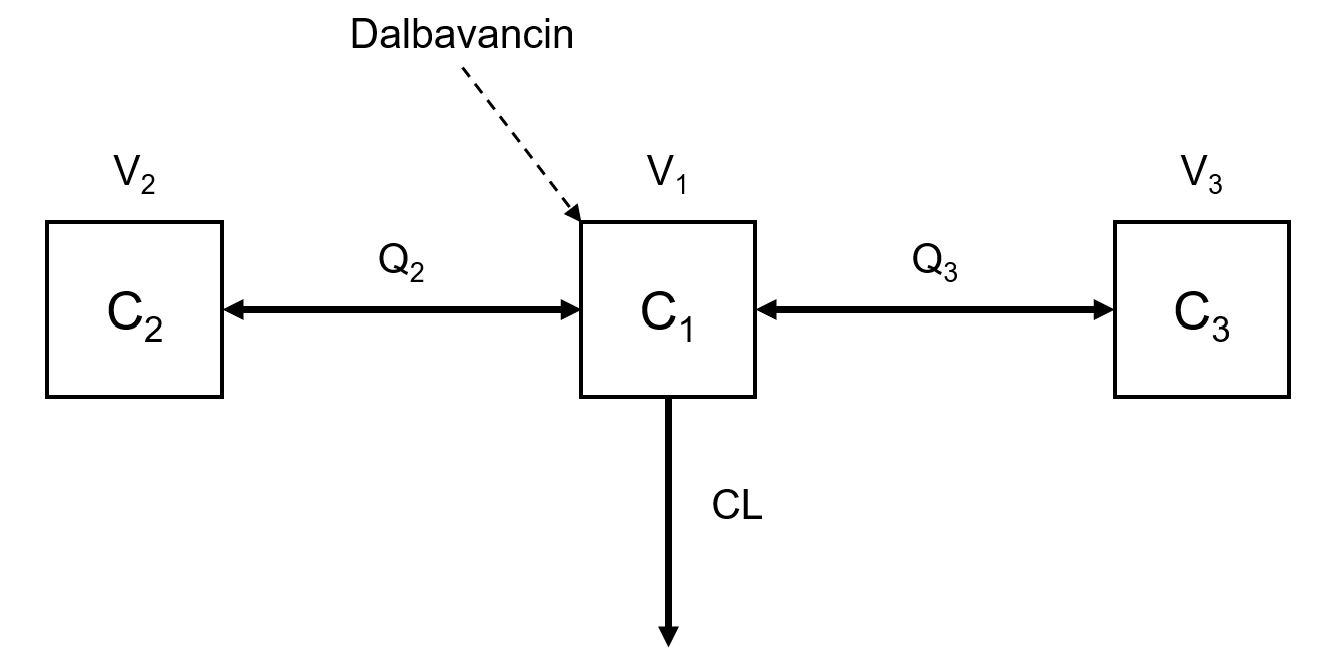
**

**Supplementary Figure S1:** Diagram summarizing the utilized population PK model.

Full model details were previously published by:

Carrothers TJ, Chittenden JT, Critchley I. Dalbavancin population pharmacokinetic modeling and target attainment analysis. *Clin Pharmacol Drug Dev*. 2020;9(1):21-31.

**NONMEM Control File**

$INPUT ID TIME AMT RATE DV WT ALB AGE CLCR MDV EVID

$DATA ..\Dalba_1000_500D14_28_Dem.csv IGNORE=#

$SUBROUTINES ADVAN6 TOL=3

$MODEL NCOMP=18

COMP=(CENTRAL DEFDOSE DEFOBS)

COMP=(TISU1)

COMP=(TISU2)

COMP=(TAT1_90)

COMP=(TAT2_90)

COMP=(TAT3_90)

COMP=(AUCAT1_90)

COMP=(AUCAT2_90)

COMP=(AUCAT3_90)

COMP=(AUCF1)

COMP=(AUC2)

COMP=(AUC3)

COMP=(TAT1_99)

COMP=(TAT2_99)

COMP=(TAT3_99)

COMP=(AUCAT1_99)

COMP=(AUCAT2_99)

COMP=(AUCAT3_99)

;------------------PK BLOCK

$PK

;IF (NEWIND.LT.2) THEN

;IFL=0

;TAD=0.0

;ENDIF

;IF (EVID.EQ.1) DTIME=TIME

;TAD=TIME-DTIME

IF(TIME.EQ.0) THEN

CMAX=0

TMAX=0

ENDIF

FU = 0.07 ; Fraction unbound dalbavancin

MICFREE90=0.06 ; SEEKING TIME AND AUC ABOVE 0.06 MCG/ML FOR DALBAVANCIN

MIC90 = MICFREE90/FU ; MIC is MIC in Total drug concentration

MICFREE99=0.25 ; SEEKING TIME AND AUC ABOVE 0.12 MCG/ML FOR DALBAVANCIN

MIC99 = MICFREE99/FU ; MIC is MIC in Total drug concentration

; --- EQN AND ESTIMATES FROM CPDD 2020:9:26 CARROTHERS

TVCL=THETA(1) * (ALB/3.7)**THETA(7) * (CLCR/100)**THETA(8) * (WT/85.5)**THETA(9)

TVV1=THETA(2) * (ALB/3.7)**THETA(10) * (WT/85.5)**THETA(11)

TVQ2=THETA(3)

TVV2=THETA(4) * (AGE/47)**THETA(12)* (ALB/3.7)**THETA(13) * (WT/85.5)**THETA(14)

TVQ3=THETA(5)

TVV3=THETA(6) * (ALB/3.7)**THETA(15) * (WT/85.5)**THETA(16)

;W1=THETA(7) ; Prop

;W2 = THETA(8)

CL=TVCL * EXP(ETA(1))

V1=TVV1 * EXP(ETA(3))

Q2=TVQ2

V2=TVV2 * EXP(ETA(2))

Q3=TVQ3

V3=TVV3 * EXP(ETA(4))

K10=CL/V1

K12=Q2/V1

K21=Q2/V2

K13=Q3/V1

K31=Q3/V3

;AUC=DOSE/CL

S1=V1 ; CONCENTRATION IN UNITS OF MG/L or MCG/ML

;-------------------DES BLOCK

$DES

CC=A(1)*FU/V1 ; CC and AUCAT will be of Free drug

CT1=A(2)*FU/V2

CT2=A(3)*FU/V3

R90C=0

R90T1 = 0

R90T2 = 0

IF(CC.GT.MICFREE90) R90C=1

IF(CT1.GT.MICFREE90) R90T1=1

IF(CT2.GT.MICFREE90) R90T2=1

R99C=0

R99T1 = 0

R99T2 = 0

IF(CC.GT.MICFREE99) R99C=1

IF(CT1.GT.MICFREE99) R99T1=1

IF(CT2.GT.MICFREE99) R99T2=1

DADT(1)=A(2) * K21 - A(1) * (K10 + K12 + K13) + A(3)*K31

DADT(2)=A(1) * K12 - A(2) * K21

DADT(3)=A(1) * K13 - A(3) * K31

DADT(4) = R90C ; TIME ABOVE MIC90 Blood TAT1

DADT(5) = R90T1 ; TIME ABOVE MIC90 TISU1 TAT2

DADT(6) = R90T2 ; TIME ABOVE MIC90 TISU2 TAT3

DADT(7) = R90C*CC ; AUC Free ABOVE MIC90 Blood AUCAT1

DADT(8) = R90T1*CT1 ; AUC Free ABOVE MIC90 Tisu1 AUCAT2

DADT(9) = R90T2*CT2 ; AUC Free ABOVE MIC90 Tisu2 AUCAT3

DADT(10)=A(1)*FU ; AUC FREE CENTRAL COMPT

DADT(11)=A(2) ; AUC TOTAL TISSUE 1 COMPT

DADT(12)=A(3) ; AUC TOTAL TISSUE 2 COMPT

DADT(13) = R99C ; TIME ABOVE MIC99 Blood TAT1

DADT(14) = R99T1 ; TIME ABOVE MIC99 TISU1 TAT2

DADT(15) = R99T2 ; TIME ABOVE MIC99 TISU2 TAT3

DADT(16) = R99C*CC ; AUC Free ABOVE MIC99 Blood AUCAT1

DADT(17) = R99T1*CT1 ; AUC Free ABOVE MIC99 Tisu1 AUCAT2

DADT(18) = R99T2*CT2 ; AUC Free ABOVE MIC99 Tisu2 AUCAT3

;----------------- ERROR MODEL ----------------------------------

$ERROR

IPRED = 0

IF(F.GT.0) IPRED=F

IRES=IPRED-DV

Y = IPRED * IPRED*EPS(1)

BLD = A(1)/V1 ; dalbavancin concentration in blood

TISU1 = A(2)/V2 ; dalbavancin concentration in compartment 2

TISU2 = A(3)/V3 ; dalbavancin concentration in compartment 3

TAT1_90=A(4) ; time above MIC90 in blood

TAT2_90=A(5) ; time above MIC90 in compartment 2

TAT3_90=A(6) ; time above MIC90 in compartment 3

AUCAT1_90 = A(7)/V1 ; AUC above MIC90 in blood

AUCAT2_90 = A(8)/V2 ; AUC above MIC90 in compartment 2

AUCAT3_90 = A(9)/V3 ; AUC above MIC90 in compartment 3

AUCF1 = A(10)/V1 ; AUC in blood

AUC2 = A(11)/V2 ; AUC in compartment 2

AUC3 = A(12)/V3 ; AUC in compartment 3

TAT1_99=A(13) ; time above MIC99 in blood

TAT2_99=A(14) ; time above MIC99 in compartment 2

TAT3_99=A(15) ; time above MIC99 in compartment 3

AUCAT1_99 = A(16)/V1 ; AUC above MIC99 in blood

AUCAT2_99 = A(17)/V2 ; AUC above MIC99 in compartment 2

AUCAT3_99 = A(18)/V3 ; AUC above MIC99 in compartment 3

REP = IREP

FUPRED = IPRED * FU ; generate IPRED concentration of free dalbavancin

;

;--------- INITIAL ESTIMATES -------------------------------------

;

$THETA (0.001, 0.0531) ;POP_CL

$THETA (1, 3.04) ;POP_V1

$THETA (0.0001, 0.288) ;POP_Q2

$THETA (1, 8.78) ;POP_V2

$THETA (0.00001, 2.11) ;POP_Q3

$THETA (1, 3.28) ;POP_V3

$THETA (-3, -0.477, 3); CL_ALB

$THETA (-1, 0.273, 2); CL_CLCR

$THETA (-1, 0.391, 2); CL_WT

$THETA (-2, -0.34, 2); V1_ALB

$THETA (-1, 0.683, 2); V1_WT

$THETA (-1, 0.486, 2); V2_AGE

$THETA (-2, -0.413, 2); V2_ALB

$THETA (-1, 0.365, 2); V2_WT

$THETA (-2, -0.551, 2); V3_ALB

$THETA (-1, 0.518, 2); V3_WT

$OMEGA BLOCK(2)

0.0489 ; PPVCL

0.0823 0.153 ; PPVV2

$OMEGA BLOCK(2)

0.0566 ; PPVV1

0.111 0.437 ; PPVV3

$SIGMA 0.0362 ; SGMA

$SIMULATION (123456) ONLYSIM SUBPROBLEMS=1
